# A pilot study of the performance of Chat GPT and other large language models on a written final year periodontology exam

**DOI:** 10.1186/s12909-025-07195-7

**Published:** 2025-05-19

**Authors:** Shaun Ramlogan, Vidya Raman, Shayn Ramlogan

**Affiliations:** 1 School of Dentistry, Faculty of Medical Sciences, The University of the West Indies, St Augustine Campus, EWMSC, Champs Fleurs, Trinidad and Tobago; 2 School of Medicine, Faculty of Medical Sciences, The University of the West Indies, St Augustine Campus, EWMSC, Champs Fleurs, Trinidad and Tobago

**Keywords:** Periodontology, Education, Clinical

## Abstract

Large Language Models (LLMs) such as Chat GPT are being increasingly utilized by students in education with reportedly adequate academic responses. Chat GPT is expected to learn and improve with time. Thus, the aim was to longitudinally compare the performance of the current versions of Chat GPT-4/GPT4o with that of final-year DDS students on a written periodontology exam. Other current non-subscription LLMs were also compared to the students. Chat GPT-4, guided by the exam parameters, generated answers as ‘Run 1’ and 6 months later as as ‘Run 2’. Chat GPT-4o generated answers as ‘Run 3’ at 15 months later. All LLMs and student scripts were marked independently by two periodontology lecturers (Cohen’s Kappa value 0.71). ‘Run 1’ and ‘Run 3’ generated statistically significantly (*p* < 0.001) higher mean scores of 78% and 77% compared to the students (60%). The mean scores of Chat GPT-4 and GPT4o were also similar to that of the best student. ‘Run 2’ performed at the level of the students but underperformed with generalizations, more inaccuracies and incomplete answers compared to ‘Run 1’ and ‘Run 3’. This variability for ‘Run 2’ may be due to outdated data sources, hallucinations and inherent LLM limitations such as online traffic, availability of datasets and resources. Other non-subscription LLMs such as Claude, DeepSeek, Gemini and Le Chat also produced statistically significantly (*p* < 0.001) higher scores compared to the students. Claude was the best performing LLM with more comprehensive answers. LLMs such as Chat GPT may provide summaries and model answers in clinical undergraduate periodontology education. However, the result must be interpreted with caution regarding academic accuracy and credibility especially in a health care profession.

## Introduction

CHAT GPT or Chat Generative Pre-trained Transformer is a Large Language Model (LLM) conversation software or Chatbot that is modified by human feedback and supervised learning. This form of artificial intelligence (AI) was developed by Open AI, Inc. (Delaware, USA) in 2022. The version Chat GPT- 4 was released in March 2023 and involved improvements in advanced reasoning, complex instructions and creativity [1]. The latest version GPT- 4o (Omni) was released in May 2024 with improvements for audio, text, image and video input producing an enhanced human–computer interaction [2].

CHAT GPT has been promoted as a more advanced user-friendly LLM research tool that allows tailored responses based on prompt engineering. While search engines retrieve and rank data from the internet, Large Language Models (LLMs) analyse, understand and generate responses from vast data on which it has been trained. This allows for the generation of seemingly original answers to questions that may be indistinguishable from those produced by humans. Moritz et al. reported that two AI plagiarism software programs (Grover and Writer Detection) were unable to determine that a machine generated paper was not generated by humans [3]. AI plagiarism software programs offer a more comprehensive detection approach looking at context and paraphrasing as opposed to tradition human plagiarism software programs which look at exact text copies without context.

Chat GPT was deemed capable of generating adequate responses for USMLE type questions in a Step 1(> 60%) but not in Step 2 (< 60%) [4]. However, in a Polish specialist medical licensing examination, CHAT GPT was able to produce a 61% score on multiple choice type questions [5]. Chat GPT has been deemed to provide fairly accurate responses to medically diverse questions as assessed by academic medical specialists [6]. However, despite high percentage of reports of correct (57.8%) and comprehensive (53.5%) answers there were instances of surprisingly incorrect answers which prompted the need to use Chat GPT with caution and to compare response to recommended data sources.

In dentistry, CHAT GPT has been used to supplement prosthodontic treatment planning and oral radiology reporting but may be limited to general information and may be cited as being at risk for medical errors and fuelling an infodemic [7, 8].

In undergraduate dental education, Chat GPT has been heralded as a transformative, interactive, inclusive, and student-centred technology that drives current pedagogical strategies [9]. Thorat et al. reported that Chat GPT aligns with an individualized student/learner centred approach which addresses the student’s need, interest and progress with autonomous student and peer learning, student engagement, motivation and tailored feedback and support. Student paper marking by AI has been proposed to have the benefits of convenience and time savings as well as consistent student feedback and quality despite the known drawbacks of ethics, legality, and privacy [10]. In comparing different forms of assessment, the performance of Chat GPT was high for multiple choice and true false questions (90–100%) while that for short essay questions was lower at 70% [11]. Chat-GPT remains one of the LLMs commonly reviewed in relation to medical education but there are many new and emerging LLMs which are gaining interest as alternatives [12, 13].

## Aim

Chat GPT may act as a potential resource for dental student education. However, the standard of response is unknown in undergraduate periodontology particularly for clinically oriented short-answer questions. Thus, the aim of this study was to generate responses via Chat GPT- 4 for a final year written exam in undergraduate periodontology and compare this to student performance. The first objective was to longitudinally repeat this process at 6 months to assess the consistency and improvement expected with this LLM. This process was also repeated at 15 months with the new version of GPT- 4o (Omni). The second objective was to compare the generated responses of other non-subscription open sourced LLMs to the students’ performance in the same exam.

## Method

This study was conducted at the School of Dentistry, The University of the West Indies, St Augustine Campus, Trinidad among final-year dental students within the DDS programme in 2023. Ethical approval was granted by the Campus Research Ethics Committee, The University of the West Indies, St Augustine Campus (CREC-SA.3172/03/2025). Individual student informed consent to participate was not required by the Campus Research Ethics Committee, The University of the West Indies, St Augustine Campus. This study relates to the following exemption regulation: FGP.P2 C 2010/2011 The University of the West Indies Policy and Procedures on Research Ethics, The School for Graduate Studies and Research on page 11 refers to"Exemptions from Review-Educational Tests and Measurements".

All students were included in the study as a pilot comparison. It was not possible to include consecutive student years due to the change in the exam questions and potential knowledge from year to year. Further, students were not individually interviewed or required to complete a questionnaire. Only the class means/medians per question were compared as a benchmark for the exam diet. The focus of the study was the performance of a non-human entity, Chat GPT and other LLMs.

The undergraduate periodontology fifth year written summative internal assessment consisted of twenty short-answer type questions over a two-hour period. The exam questions were mapped and benchmarked to the learning objectives providing balanced coverage of the periodontology syllabus of the clinical years. Guided by Bloom’s taxonomy for the cognitive domain, questions discerned knowledge, comprehension, application, analysis, synthesis, and evaluation learning objectives. This written exam did not include clinical photographs or radiographs as these may have been challenging to interpret by Chat GPT. However, clinical scenarios and narratives were included. Each student was asked to identify five easy and five difficult questions based on individual opinion. This was simply indicated on the examination script by the students and reported as a distribution frequency.

Model answers and marking schemes were developed by both lecturers in periodontology. Both lecturers independently marked all scripts and the final percentage mark was determined from an average of the assigned scores. The inter-rater agreement for the two periodontology lecturers was good with a significant (*p* < 0.001) Cohen’s Kappa (for two raters) statistical value of 0.71 when comparing individual questions for all scripts.

Responses for the Periodontology written exam were obtained from the subscription-based version of Chat GPT 4 (Chat Plus Subscription, Chat GPT- 4, Open AI, San Francisco, USA) in September 2023 as ‘Run 1’. The request/prompt for Chat GPT- 4 included parameters such as the nature of the exam, the number of marks assigned and the number of lines/words available to respond to each question. The exam prompt with the third exam question as an example was as follows:

*‘For all questions respond exactly as a final (5th) year dental student writing their periodontology written examinations. (It is a 2 h exam consisting of 20 short answer questions).*

The third question below is worth 6 marks and should be done in roughly 77 words (as 7 lines on an A4 writing paper is given):

Give the vertical furcation classification as defined by Tarnow and Fletcher? How would you manage a Grade II, Class B furcation defect on a lower right first molar?’

The generated Chat GPT- 4 answers were hand transcribed to a hard copy by a research assistant and randomly placed with the students’ scripts to ensure blinding for both markers. Six months later in March 2024, a second generated answer paper, ‘Run 2’ was obtained from Chat GPT- 4 using identical prompt protocols.

Answers for the written exam were also generated from the latest model GPT- 4o (OpenAI, May 2024) as ‘Run 3’. Other non-subscription-based LLMs were selected which were openly accessible to the public and capable of execution on a home-based computer within the parameters of memory or computer processing unit (CPU) power. These included the following 10 selected models: (1) C4ai (CohereForAI) (Version: c4ai-command-r-plus August 2024; Cohere, Toronto, Canada), (2) Claude (Version: Claude3.5-sonnet October 2024; Anthropic, San Francisco, California, USA), (3) DeepSeek (Version: DeepSeek-R1 January 2025; DeepSeek, Hangzhou, Zhejiang, China) (4) Gemini (Version: Gemini 2.0 flash December 2024; Google, Mountain View, California, USA), (5) Mistral NeMo (Version: Nemo-Instruct- 2407 July 2024; NVIDIA, Santa Clara, California, USA and Mistral AI, Paris, France), (6) Le Chat- Mistral (Version: Le Chat February 2024; Mistral AI, Paris, France), (7) Llama- 3.1 (Version: Llama- 3.1-Nemotron- 70B-Instruct July 2024; NVIDIA, Santa Clara, California, USA and Meta Platforms Inc., California, USA), (8) Llama- 3.3 (Version: Llama- 3.3 - 70B-Instruct December 2024; Meta Platforms Inc., California, USA), (9) Phi (Version: phi- 4-Q4_K_M December 2023; Microsoft -phi series, Redmond, Washington, USA). (10) Qwen (Version: Qwen2.5 - 72B September 2024; Alibaba Cloud, Hangzhou, China). Answers from the above 10 LLMs and GPT- 4o were generated in January 2025 according to the same protocols. Both examiners marked the generated scripts and the average of the two examiners were used as the assigned mark per question.

Marks were entered into SPSS (IBM SPSS Statistics 30.0) for t-test statistical analyses (significance p < 0.05) comparing the exam scores means of students to the exam scores of Chat GPT and other LLM with graphical representation. A review of the answers generated for ‘Run 1’, ‘Run 2’ and ‘Run 3’ was completed by both lecturers.

## Results

Twenty-two students, 86.4% (*n* = 19) of whom were female and 13.6% (*n* = 3) of whom were male, with a mean age of 26.1 years (standard deviation 2.9) in their final year participated in the written exam. The student mean percentage score for the written exam was 60% (standard deviation 12.6; range 33 to 78). Based on the opinions of the students, questions 2 (*n* = 14), 5 (*n* = 16), 10 (*n* = 16), 12 (*n* = 8) and 18 (*n* = 8) were reported most frequently as the easiest. Conversely, questions reported with the highest frequency of difficulty were 8 (*n* = 13), 9 (*n* = 12), 15 (*n* = 14), 16 (*n* = 8) and 17 (*n* = 9).

**Fig. 1:**
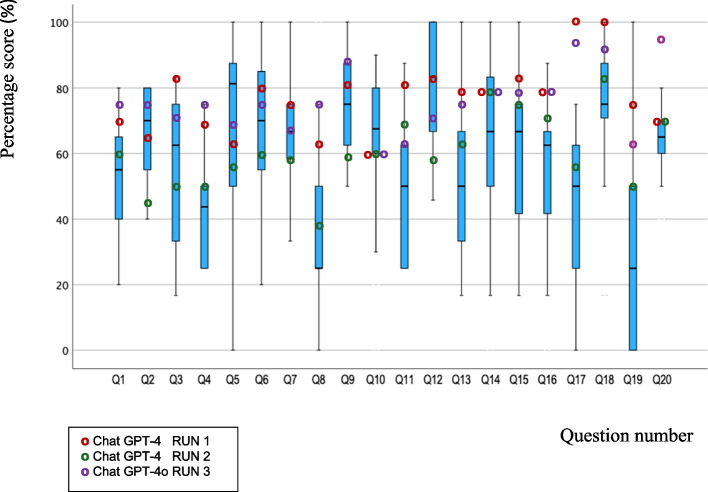
Boxplot of students’ percentage scores with overlays of mean percentage scores for Chat GPT: RUN 1, RUN 2 & RUN 3 per question

The mean percentage score for the written exam derived from Chat GPT- 4, ‘Run 1’ was 78% (standard deviation 10.8) (Table 1). ‘Run 1’ produced answers that were scored above the students’ median score for 16 questions out of the 20 questions (Fig. 1). There were two questions for which ‘Run 1’ produced full scoring answers (questions 17 and 18). For all questions, the ‘Run 1’ score was 60% or greater. For questions 2, 5 and 10, the score for ‘Run 1’ was less than the corresponding median student score. Questions 2, 5 and 10 covered topics of periodontal disease screening and classification. Conversely, ‘Run 1’ produced a full score in question 17 for which the students’ median score was only 50%.

**Table 1:** Comparison of mean percentage score of students to Chat GPT- 4/GPT4o and other LLMs

Source	Mean Percentage Score (%)	Standard Deviation	Significance t-test (*p* values) Comparison to Students’ Scores
Student	60	12.6	-
Subscription Based- Open AI Chat GPT- 4/GPT4o			
Run 1 (GPT- 4)	78	10.8	< 0.001*
Run 2 (GPT- 4)	62	11.4	0.52
Run 3 (GPT- 4o)	77	10.0	< 0.001*
Non-Subscription Based- Other LLM			
C4a1	66	14.3	0.09
Claude	87	11.4	< 0.001*
DeepSeek	78	13.0	< 0.001*
Gemini	78	13.9	< 0.001*
LeChat	72	14.1	0.005*
Llama 3.1	63	14.4	0.22
Llama 3.3	55	12.8	0.61
Mistral	49	12.0	0.02#
Phi	68	20.6	0.09
Quen	62	12.8	0.49

^#^significantly lower

^*^significantly higher

The mean percentage score for the written exam derived from Chat GPT- 4, ‘Run 2’ was 62% (standard deviation 11.4) (Table 1). In this second attempt of Chat GPT- 4 at 6 months later, ‘Run 2’ produced scores above the students’ median score for only 11 questions out of the 20 questions (Fig. 1). Chat GPT- 4 scored less than 50% on questions 2 and 8 which covered topics on periodontal disease screening and gingival recession classification. There were no full scoring (100%) answers in ‘Run 2’ as there was in ‘Run 1’.

In ‘Run 3’, the mean percentage score for the written exam derived from GPT- 4o (Omni) was 77% (standard deviation 10.0) (Table 1). In this ‘Run 3’ attempt which was about 15 months after ‘Run 1’, there were comparable results to ‘Run 1’ with all questions scoring 60% and above (Fig. 1). ‘Run 3’ produced scores above the students’ median score for 17 questions out of the 20 questions. However, there were also no full scoring (100%) answers in ‘Run 3’ as there was in ‘Run 1’. ‘Run 3’ scored less than the median student score for questions 5, 10 and 12 which covered topics of periodontal disease classification and antiseptic mouthwash.

Both ‘Run 1’ and ‘Run 3’ produced a statistically significantly (t-test; *p* < 0.001) higher mean percentage score when each was separately compared to that of the students’ mean percentage score (Table 1). However, the mean percentage score of ‘Run 2’ failed to show statistically significant difference (t-test; *p* = 0.52) from the students’ mean score (Table 1). Additionally, both ‘Run 1’ and ‘Run 3’ produced a statistically significantly (t-test; *p* < 0.001) higher mean percentage score when each was separately compared to that of ‘Run 1’. There was no statistically significant difference (t-test; *p* = 0.70) between the mean percentage score of ‘Run 1’ and ‘Run 2’.

With regards to questions 5 and 10 which addressed the current knowledge of the latest classification for periodontal disease (Staging and Grading), all three runs (‘Run 1’, ‘Run 2’, ‘Run 3’) underperformed in relation to the students (Fig. 1). ‘Run 1’ also underperformed in relation to the students in question 2 due to a lack of detail for Basic Periodontal Examination (BPE) codes and higher order analysis of the clinical scenario [14]. In general, for ‘Run 1’ full marks were not attained due to the use of older terminology, lack of detail and lack of explanation for some of the answers. This older terminology included the former periodontology classification groups of Chronic and Aggressive Periodontitis.

In ‘Run 2’, there were more incomplete answers lacking details which were previously given in ‘Run 1’ (Table 2; refer to question 17). There were also wrong and inaccurate answers in ‘Run 2’ which were not given in ‘Run 1’ (Table 2; as shown in question 3). There continued to be a problem with defining the new periodontology terminology in relation to gingival recession defects as viewed in question 8 for both ‘Run 1’ and ‘Run 2’ (Table 2). For question 2, the BPE codes were correctly identified in ‘Run 1’ but were incorrectly identified in ‘Run 2’ (Table 2).

**Table 2:** Comparison of Chat GPT- 4 Run 1 to Run 2 with excerpt questions and responses

Question	Chat GPT- 4 RUN 1	Chat GPT- 4 RUN 2	Comments
2. What is the management of a patient with these BPE codes? 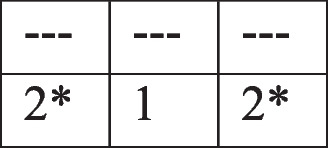	..indicate gingivitis (Code 1&2) with * furcation involvement	..* indicating bleeding on probing	RUN 2 incorrectly identifies * as bleeding on probing and doesn’t mention furcation involvement or what Code 1 or 2 represents
3. Give the vertical furcation classification as defined by Tarnow and Fletcher	..defines Class A as 1–3 mm, Class B as 4–6 mm and Class C As > 6 mm	..includes Grade I (incipient), Grade II (moderate) and Grade III through and through	Incorrect information ascribed to the vertical classification (Tarnow and Fletcher) in RUN 2
8. How would you manage a gingival recession defect of RT3?	..management of.. right upper third molar (RT3) depends on the severity… aesthetics and hypersensitivity	..includes surgical intervention such as CT graft or GTR to cover exposed root surface and increase soft tissue volume	RUN 1 incorrectly identified what RT3 meant (new classification). RUN 2 didn’t define this parameter and gave incorrect options
17. What is the odds ratio for periodontitis in a diabetic?	..in diabetics is estimated to be 2–3 times higher compared to non-diabetics suggesting an increased risk	..in diabetics is sig higher vs non-diabetics indicating a strong association between diabetes and the risk of periodontal disease	Less detail in RUN2 compared to RUN1. Numerical value of odds ratio left out in RUN 2

‘Run 3’ was able to correctly identify the gingival recession type, RT3 in question 8 unlike ‘Run 1’ and ‘Run 2’. However, overall answers in ‘Run 3’ were similar to ‘Run 1’ despite the separation of a 15 month timeline.

**Fig. 2:**
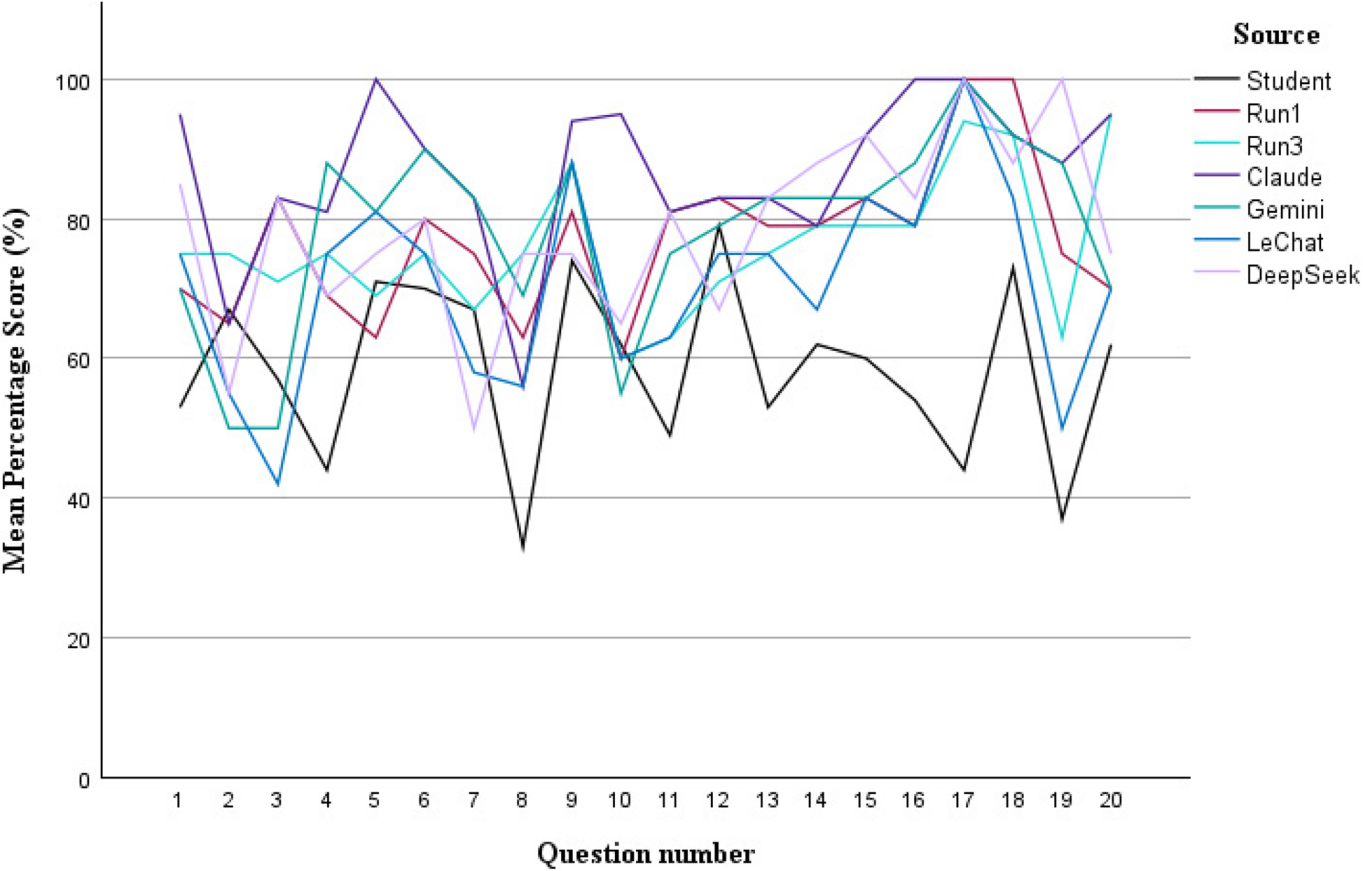
Mean percentage score by question for statistically significant LLMs compared to students

The comparison of the other non-subscription Large Language Models (LLMs) to student performance in the written exam was also shown in Table 1. All models except Llama 3.3 and Mistral performed equally or better than the students in the written exam. The models which produced a statistically significantly (t-test) higher mean percentage score compared to the students were Claude (*p* < 0.001), DeepSeek (*p* < 0.001), Gemini (*p* < 0.001) and Le Chat (*p* = 0.005). These better performing models together with ‘Run 1’ and ‘Run 3’ are graphically represented across the 20 exam questions in relation to the student performance (Fig. 2).

Claude produced statistically significantly (t-test) higher mean scores compared to both ‘Run 1’ (*p* = 0.003) and ‘Run3’ (*p* = 0.002). However, Le Chat generated statistically significantly (t-test) lower mean scores compared to both ‘Run 1’ (*p* = 0.04) and ‘Run3’ (*p* = 0.04). Both DeepSeek and Gemini did not produce statistically significant differences (t-test) in the mean scores from either ‘Run 1’ (DeepSeek *p* = 0.55; Gemini *p* = 0.61) or ‘Run3’ (DeepSeek *p* = 0.44; Gemini *p* = 0.47).

## Discussion

Both Chat GPT- 4 ‘Run 1’ and GPT- 4o ‘Run 3’ attained statistically significantly (t-test; p < 0.001) greater percentage scores (‘Run 1′ 78%, ‘Run 3′ 77%) compared to students’ mean percentage (60%) score in this clinically oriented short-answer questions format of the final written periodontology exam. The performance of Chat GPT- 4/GPT4o in this format exam was similar to that reported in the literature for short essay questions [11]. The Chat GPT- 4/GPT4o scores were also equivalent to that of the best performing student in the class. When considering only ‘Run 1’ or ‘Run 3’, Chat GPT- 4 or the updated GPT- 4o may be assumed to be a good resource that may aid students in providing information summaries and model answers.

However, the major limitations are the generalization of answers, lack of detail and the use of outdated terminology. Some of the information related to the classification of periodontal disease was dated due to the use and access of older data sources by Chat GPT- 4/GPT- 4o. The published knowledge cutoffs for Chat GPT- 4 and GPT- 4o models are December, 2023 and October, 2023 respectively [15]. Although the new periodontal classification was published in 2018, five years earlier to these cutoffs, the information remains inaccurate or outdated [16]. This may indicate the drawback of Chat GPT- 4/GPT- 4o to provide current information with advancing academic and clinical standards in dentistry. Students deemed questions 5 and 10 as easier and performed better in these questions on periodontal disease classification than Chat GPT- 4/GPT- 4o. This may be due to better clinical pedagogical strategy and motivation by the lecturers in these current topics which reinforces the value of the human element in clinical education.

Chat GPT- 4, ‘Run 2’ underperformed compared to both Chat GPT- 4, ‘Run 1’ and GPT- 4o, ‘Run 3’. This was an unexpected outcome as it was assumed that this LLM would produce either comparable or improved answers with time through progressive learning. However, Chat GPT- 4, ‘Run 2’ surprisingly produced some incorrect information that was not generated in ‘Run 1’. There was also a reduction in detailed information in ‘Run 2’ compared to ‘Run 1’. Errors at the discipline speciality level by Chat GPT were also previously reported in the literature [6]. Open AI had suggested that while Chat-GPT- 4 was an improvement on previous versions, it may still be prone to ‘hallucinations’ and reasoning errors [17]. Students reported difficulty with question 8 on gingival recession classification and performed weakly on this question as it also required a higher order of analysis. Chat GPT- 4 in ‘Run 2’ was unable to interpret the clinical scenario correctly in question 8 and scored below 40% while ‘Run 3’ performed better at 75%.

A lack of detail may have been a random outcome as Chat GPT attempts to deliver original text in each response. This generated originality may be undetectable by plagiarism software as reported by other authors [3]. Other possibilities include inherent LLM limitations at the level of the Chat GPT- 4 provider which may be constrained by variations in online traffic, availability of datasets and support resources. At the time of the study, the current version of the subscription-based Chat GPT 4 was used in this study to facilitate the best possible use of this LLM technology. Inaccuracies may also be the result of biased data input sources which may skew the generated responses [9]. The limitation in word count was applied equally to both ‘Run 1’ and ‘Run 2’ of Chat GPT- 4 and thus was not a confounding factor. However, in a non-examination scenario, Chat GPT- 4 may generate more complete responses if it is not limited by word count.

Among the other investigated LLMs, Claude, DeepSeek, Gemini and Le Chat performed statistically significantly better than the students. DeepSeek and Gemini performed at the level of Chat GPT- 4/GPT- 4o while Le Chat performed below the level of Chat GPT- 4/GPT- 4o. Claude performed statistically significantly better than Chat GPT- 4/GPT- 4o as the best overall performing LLM with more comprehensive answers. Claude gave the best responses to the questions on periodontal disease classification but not on gingival recession classification. This pilot study may potentially indicate the value of Claude in generating responses for clinically oriented short answer questions in periodontology. The non-subscription LLMs may have been limited in their full performance capacity as they were openly accessible to the general public. Thus some reservation should be applied to this comparative analysis of the non-subscription LLMs.

A study limitation was the use of one class of students as the reference standard. The performance of students may vary from year to year and this one diet of exam results may not be reflective of all students in periodontology. Additionally, as all students were included and a power calculation was not attempted, the results may be deemed as a pilot study. A further limitation may be the applicability of this study to only (1) clinically oriented short answer questions (format) and (2) periodontology (subject). Chat GPT- 4 was used to generate answers without further prompting or interaction. This would have restricted the refinement and correction of answers expected in an ongoing chat with a LLM.

## Conclusion

Chat GPT- 4/GPT- 4o, Claude, DeepSeek and Gemini may generate adequate clinically oriented short answers at the dental undergraduate level in periodontology. Variable responses as demonstrated by Chat GPT- 4 over time and inaccuracies stemming from outdated information, hallucinations and other inherent LLM limitations may impact on health care professional training. LLMs may be better suited for use as an adjunctive educational aid to delivered courses guided by the required tutors due to the necessity for human oversight and validation. A more productive and dynamic use of this LLM would involve further interactive enquiry and critical engagement of the generated content.

Future work may look at the changes in the longitudinal responses of the other high performing non subscription LLMs in this study. As LLMs evolve and develop, the application to more complex data such as clinical photos and radiographs may also be investigated. Finally, further investigations may also look at the impact of use of LLMs in assessments, identification of plagiarism and the implications for academic integrity.

## Data Availability

The datasets generated and/or analysed during the current study are not publicly available but are available from the corresponding author on reasonable request.
